# Symptom severity in autism spectrum disorder is related to the frequency and severity of nausea and vomiting during pregnancy: a retrospective case-control study

**DOI:** 10.1186/s13229-018-0223-7

**Published:** 2018-06-19

**Authors:** Andrew J. O. Whitehouse, Gail A. Alvares, Dominique Cleary, Alexis Harun, Angela Stojanoska, Lauren J. Taylor, Kandice J. Varcin, Murray Maybery

**Affiliations:** 1 Telethon Kids Institute, The University of Western Australia, 100 Roberts Rd, Subiaco, WA 6009 Australia; 2 Department of Psychology, Institute of Psychiatry, Psychology and Neuroscience, King’s College London, 16 De Crespigny Park, London, UK; 3 School of Psychological Science, The University of Western Australia, 35 Stirling Highway, Crawley, 6009 Australia; 4 Telethon Kids Institute, University of Western Australia, 100 Roberts Road, Subiaco, WA 6008 Australia

## Abstract

**Background:**

Nausea and vomiting during pregnancy (NVP) is thought to be caused by changes in maternal hormones during pregnancy. Differences in hormone exposure during prenatal life have been implicated in the causal pathways for some cases of autism spectrum disorder (ASD). However, no study has investigated whether the presence and severity of NVP may be related to symptom severity in offspring with ASD.

**Methods:**

A large sample of children with ASD (227 males and 60 females, aged 2 to 18 years) received a clinical assessment, during which parents completed questionnaires regarding their child’s social (Social Responsiveness Scale, SRS) and communication (Children’s Communication Checklist–2nd edition, CCC-2) symptoms. Parents also reported on a 5-point scale the frequency and severity of NVPs during the pregnancy of the child being assessed: (1) no NVP during the pregnancy, (2) occasional nausea, but no vomiting, (3) daily nausea, but no vomiting, (4) occasional vomiting, with or without nausea, and (5) daily nausea and vomiting.

**Results:**

Impairments in social responsiveness in offspring, as indexed by SRS total score, significantly increased as a function of the frequency and severity of their mothers’ NVP, as did the level of language difficulties as indexed by the Global Communication Composite of the CCC-2.

**Conclusions:**

The strong, positive association between increasing frequency and severity of NVP and ASD severity in offspring provides further evidence that exposure to an atypical hormonal environment during prenatal life may affect neurodevelopment and contribute to the ASD phenotype. Given that the measure of NVP symptoms in the current study was based on retrospective recall, replication of this finding is required before strong conclusions can be drawn.

**Electronic supplementary material:**

The online version of this article (10.1186/s13229-018-0223-7) contains supplementary material, which is available to authorized users.

## Background

Autism spectrum disorder (ASD) is the collective term for developmental disabilities characterized by impairments in social interaction and verbal and nonverbal communication and by repetitive patterns of behavior. While the biological pathways contributing to ASD remain unclear, current consensus is for a multifactorial etiology, incorporating a constellation of genetic risk variants that may interact with environmental factors [1]. The in utero hormone environment is critical to development of the structure and function of the central nervous system, and there is increasing evidence that prenatal endocrine factors may also play a key role in the etiology of some cases of ASD [2]. A particular focus of previous research has been on fetal exposure to elevated levels of androgens, measured via sampling of amniotic fluid during the second trimester [3] and umbilical cord blood at birth [4]. Exposure to elevated concentrations of prenatal androgens has been found to be related to a diagnosis of clinical ASD in offspring [3], as well as autistic-like traits in the general population [5, 6], particularly communication difficulties [7, 8]. While it is important to highlight that these findings have not always been replicated [4, 9], further support for the potential role of the prenatal hormone environment in the etiological pathways contributing to ASD has been observed through associations with factors known to influence maternal hormone status, such as maternal age at menarche [10], pre-pregnancy BMI [11], and infertility and its treatments [10, 12]. This evidence strongly supports the further examination of how prenatal endocrine factors may contribute to ASD.

Nausea and vomiting during pregnancy (NVP) is experienced by approximately 70 to 80% of pregnant women [13]. The timing, frequency, and duration of NVP varies between women and, in its most extreme form (hyperemesis gravidarum), is characterized by severe and persistent nausea and vomiting that results in a significant loss in body weight [14]. While NVP is known to involve a heritable component [15, 16], the biological mechanisms underpinning the maternal experience of severe NVP are thought to involve the dysregulation of maternal human chorionic gonadotropin (hCG), thyroid hormones (particularly thyroxine and thyroid-stimulating hormone), and estrogens [14, 17]. There are a number of reasons to hypothesize a link between NVP and the ASD phenotype among offspring. First, women who experience hyperemesis gravidarum have a greater risk of having a child with behavioral and neurodevelopmental conditions [18, 19]. While no study has been sufficiently powered to investigate offspring risk for ASD, an increased risk has been observed for common neurodevelopmental comorbidities for ASD, such as intellectual disability, developmental language disorder, and attentional deficit hyperactivity disorder [20]. Second, differential levels of hCG during pregnancy have been associated with later ASD in offspring. A recent study of Californian state records examined the association between hCG levels during pregnancy (collected for routine screening between 15 and 20 weeks’ pregnancy) and the prevalence of ASD among offspring [21]. An increased risk of offspring ASD was identified for women with the highest and lowest decile of hCG concentration distribution, suggesting a link between atypical hCG regulation and offspring ASD. Third, a number of large studies have identified an association between maternal autoimmune thyroid disease during pregnancy and risk for ASD [22, 23], with a recent meta-analysis reporting a pooled odds ratio of 1.29 (95% confidence interval 1.14–1.45) [24].

Considered together, the accumulating body of research presents the hypothesis that an atypical profile of pregnancy-related hormones may be involved in the etiological pathway for some cases of ASD, and that this may be indexed by NVP. The current study provides the first investigation of whether maternal NVP relates to symptom severity in children with ASD. Given evidence linking the experience of any nausea or vomiting symptoms during pregnancy to hormone dysregulation [25], the study examined ASD symptomatology in relation to the full severity of NVP symptoms. Based on previous findings linking NVP to neurodevelopmental delay and disorder [20], and the association between hormones implicated in NVP and risk for offspring ASD [21], we hypothesized that increased frequency and severity of maternal NVP would relate to more severe ASD symptoms among children.

## Methods

### Participant sample

Participants were part of the Western Australian Autism Biological Registry, which is an ongoing study of children with ASD and their families taking place at the Telethon Kids Institute in Perth, Western Australia [26]. Participants were recruited via newspaper advertisements, and children with a clinical diagnosis of an ASD were included in the study. In Western Australia, diagnosis of ASD is strictly regulated and obtained by consensus following a multidisciplinary assessment by a team comprising a pediatrician, clinical psychologist, and speech pathologist [27]. Ethics approval for the WAABR was granted by the Human Ethics Committee at Princess Margaret Hospital for Children in Perth, Western Australia. The primary caregiver provided informed consent to be a part of the study. The sample included 227 males and 60 females with ASD aged 2 to 18 years at the time of data collection (mean = 8.28 years; SD = 3.89 years).

### Procedure

Parents and their child(ren) with ASD were invited to the Telethon Kids Institute for a face-to-face assessment. One week prior to this assessment, parents were mailed several questionnaires, which they were asked to complete and return to the research team at the time of the research appointment.

### Obstetric history

A primary caregiver completed a family history questionnaire, which included questions about demographic characteristics and the social and medical history of the family. A section in the questionnaire asked questions about the history of the pregnancy that resulted in the child with ASD, including questions about any obstetric complications that occurred. The child’s biological mother was specifically asked to complete this section. Fetal growth restriction was defined as a birthweight lower than the 10th percentile for gestational age, according to Australian norms [28]. A specific question in the questionnaire asked whether the mother experienced nausea and/or vomiting during pregnancy, and respondents were asked to answer on a 5-point scale: (1) no nausea or vomiting, (2) occasional nausea, but no vomiting, (3) daily nausea, but no vomiting, (4) occasional vomiting, with or without nausea, and (5) daily nausea and vomiting.

### ASD symptom severity

The primary caregiver was asked to complete two questionnaires relating to their child’s ASD symptomatology.

#### Social Responsiveness Scale–Parent Report Form

The Social Responsiveness Scale (SRS) is a 65-item caregiver report questionnaire designed to measure social behavior, language, and repetitive behavior/restricted interests in children [29]. Each item asks about a social behavior, which respondents rate on a scale from 0 (never true) to 3 (almost always true). In addition to the SRS total score, there are five subscales assessing social awareness, social cognition, social communication, social motivation, and autistic mannerisms. Analyses were conducted on the raw scores for the total scale and for the subscales since these scores were normally distributed, whereas the distributions for norm-based T scores were skewed, containing a substantial proportion of cases scoring at the maximum T scores (*T* ≥ 90). Higher scores indicate more pronounced ASD symptoms. SRS total scores were available for 282 of the cases (223 males, 59 females).

#### Children’s Communication Checklist–2nd edition.

The Children’s Communication Checklist–2nd edition (CCC-2) [30] is a 70-item parent report questionnaire designed to screen for communication difficulties in children with phrase speech. The scale is comprised of ten subscales that measure general communication difficulties (speech, syntax, semantics and coherence), pragmatic language (inappropriate initiation, stereotyped language, use of context, and nonverbal communication), and behaviors commonly associated with ASD (social behavior and interests). Age-referenced standard scores with a mean of 10 (SD = 3) can be derived for each subscale for participants aged between 4 and 18 years. A composite measure of communication ability is also obtained (Global Communication Composite, GCC), which is a summed score of the eight subscales relating to general communication difficulties and pragmatic language. On all scales, lower scores equate to greater difficulties. GCC scores were available for 209 participants (166 males, 43 females) aged 4–18 years.

### Statistical analyses

SRS raw and CCC-2 standard scores were checked for outliers using the criterion |*z*| ≥ 3.29. No more than two outliers were identified for any single measure. These scores were removed prior to the further analysis. The principal analyses consisted of two ANCOVAs conducted on the SRS total scores and CCC-2 GCC standard scores, with four covariates: paternal age, maternal age, child sex, and the child’s age at assessment (which either differed across NVP groups or correlated with the key symptom variables; see below). In follow-up analyses, linear, quadratic, cubic, and quartic trends were calculated for the NVP factor. A power analysis (Additional file 1) indicated that the study was not sufficiently powered (*d* < 0.8) to examine the data separately by sex, due to the relatively small number of females (*n* = 60). Given the increasing interest in sex differences in ASD [31], the sex-specific descriptive statistics for the SRS and CCC-2 (total/GCC and subscales) have been included as Additional file 1. However, we urge caution in the interpretation of these data given the concerns pertaining to low statistical power and multiple comparisons.

## Results

### Demographic characteristics of NVP groups

Table 1 provides descriptive statistics for the five groups defined by frequency and severity of the mothers’ NVP. The distribution of cases across the NVP groups differed for males and females, *χ*^2^(4) = 12.24, *p* < .05 (see Table 1). In particular, the most severe form of NVP was experienced by 31.67% of the mothers of females but by only 17.62% of the mothers of males, with *χ*^2^(1) = 5.73, *p* < .05, for this comparison. In the current sample, the distribution of cases across the NVP categories did not depend on whether the mother had preeclampsia during pregnancy, *χ*^2^(4) = 4.58, *p* = .33, or whether, at birth, the child was assisted in breathing, *χ*^2^(4) = 2.57, *p* = .63, or required special nursery care, *χ*^2^(4) = 4.07, *p* = .40. The NVP group was also unrelated to fetal growth restriction in males, *χ*^2^(4) = 2.15, *p* = .71, or females, *χ*^2^(4) = .52, df = 4, *p* = .97. Comparisons using one-way ANOVA also showed that the NVP groups did not differ on age at time of data collection, *F*(4,282) = 1.14, *p* = .34, ${\eta}_p^2$ = .016, age when ASD was diagnosed, *F*(4,270) = 1.55, *p* = .19, ${\eta}_p^2$ = .022, and gestational age at birth, *F*(4,274) = 0.76, *p* = .56, ${\eta}_p^2$ = .011. However, the NVP groups did differ significantly on paternal age at conception of the child, *F*(4,279) = 2.91, *p* < .05, ${\eta}_p^2$ = .040, and the analysis comparing the groups on maternal age at conception yielded a marginally significant result, *F*(4,281) = 2.02, *p* = .09, ${\eta}_p^2$ = .028 (see Table 1 for means).

**Table 1:** Group descriptive statistics for children with ASD as a function of the frequency and severity of their mothers’ nausea and vomiting during pregnancy

	No nausea and vomiting during pregnancy (*n* = 58)	Occasional nausea but no vomiting (*n* = 71)	Daily nausea but no vomiting (*n* = 58)	Occasional vomiting with or without nausea (*n* = 41)	Daily nausea and vomiting (*n* = 59)
Child sex (%, *n*)^a^					
Male	20.7 (47)	28.6 (65)	18.9 (43)	14.1 (32)	17.6 (40)
Female	18.3 (11)	10.0 (6)	25.0 (15)	15.0 (9)	31.7 (19)
Maternal preeclampsia during pregnancy (%, *n*)					
No	20.9 (55)	24.0 (63)	20.5 (54)	13.3 (36)	21.3 (56)
Yes	12.5 (3)	33.3 (8)	16.7 (4)	25.0 (3)	12.5 (3)
Fetal growth restriction (%, *n*)					
No	19.5 (43)	24.4 (54)	19.9 (44)	15.8 (35)	20.4 (45)
Yes	15.8 (6)	26.3 (10)	18.4 (7)	13.2 (5)	26.3 (10)
Child required breathing assistance after birth (%, *n*)					
Not required	20.2 (50)	25.9 (64)	21.1 (52)	13.4 (33)	19.4 (48)
Required	21.1 (8)	18.4 (7)	15.8 (6)	18.4 (7)	26.3 (10)
Child required special nursery care after birth (%, *n*)					
Not required	21.2 (47)	23.0 (51)	19.4 (43)	15.8 (35)	20.7 (46)
Required	17.7 (11)	30.6 (19)	24.2 (15)	8.1 (5)	19.4 (12)
Child’s gestational age at birth (weeks)					
Mean (SD, *n*)	38.75 (2.42, 55)	38.61 (2.56, 70)	38.25 (2.86, 56)	38.71 (2.16, 41)	39.02 (1.75, 57)
Maternal age at conception (years)^b^					
Mean (SD, *n*)	31.74 (5.61, 58)	32.25 (5.11, 71)	31.73 (4.99, 58)	29.80 (5.45, 41)	30.46 (4.91, 58)
Paternal age at conception (years)					
Mean (SD, *n*)	34.12 (5.29, 57)	35.26 (6.23, 70)	33.33 (6.17, 58)	31.33 (6.10, 41)	33.34 (6.34, 58)
Child age at time of ASD diagnosis (years)					
Mean (SD, *n*)	4.26 (2.88, 58)	4.69 (2.74, 65)	4.25 (2.84, 57)	5.05 (3.01, 38)	5.42 (3.67, 57)
Child age at time of data collection (years)					
Mean (SD, *n*)	7.94 (3.31, 58)	7.67 (3.76, 71)	8.52 (3.63, 58)	9.11 (4.35, 41)	8.54 (4.41, 59)

^a^Chi-square analysis, *p* < .05

^b^One-way ANOVA, *p* < .05

### Relationships of ASD symptom severity measures to demographic variables

SRS total and CCC-2 GCC scores were correlated with the child’s age at assessment, maternal and paternal age at conception of the child, and child’s gestational age at birth. SRS total score correlated significantly with both the child’s age at assessment, *r* = 0.21, *p* < .001, and maternal age, *r* = − .16, *p* < .01, and CCC-2 GCC scores also correlated with the child’s age at assessment, *r* = − .44, *p* < .001. No other correlations were significant. Neither of the two key symptom measures differed significantly as a function of whether the mother had preeclampsia during pregnancy, larger *t*(207) = 1.26, *p* = .21, nor whether the newborn was assisted in breathing at birth, larger *t*(278) = .37, *p* = .71, required special nursery care, larger *t*(277) = .77, *p* = .44, or was of restricted growth at birth, larger *t*(252) = .73, *p* = .19.

### ASD symptom severity as a function of maternal NVP

**Fig. 1:**
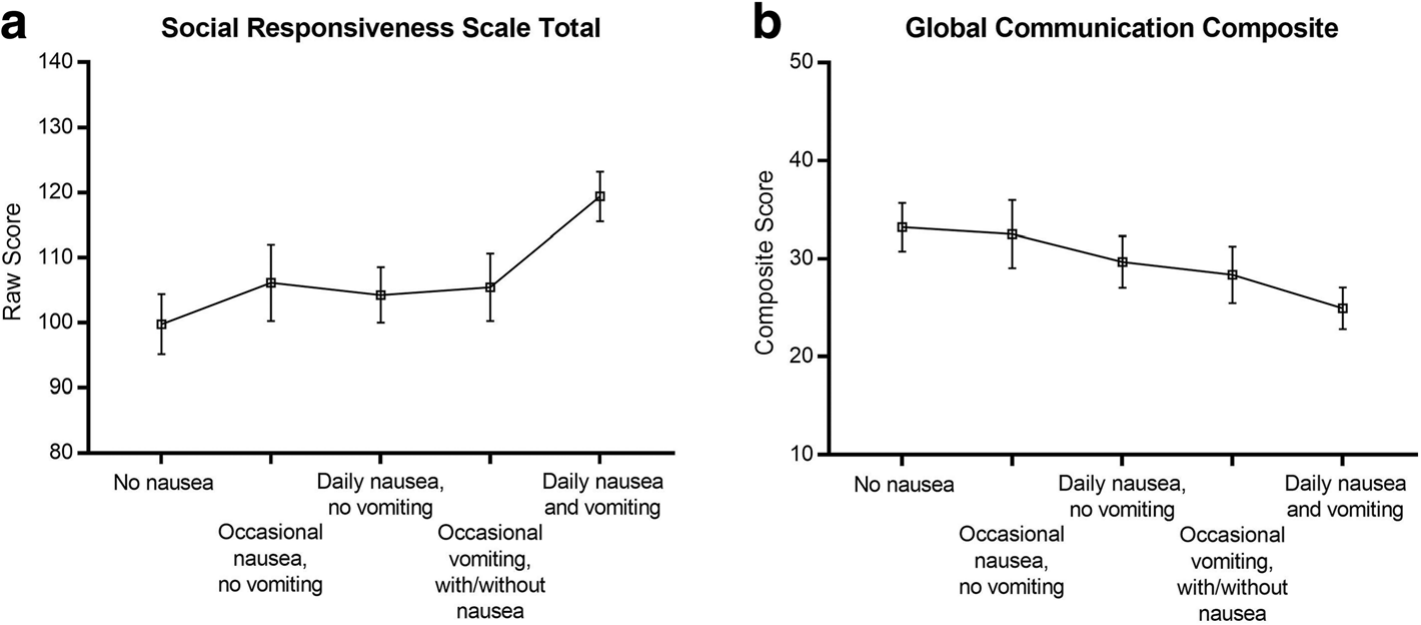
Mean scores on **a** the SRS total score and **b** the CCC-2 GCC, as a function of maternal nausea and vomiting during pregnancy. Error bars represent standard errors of the mean

Table 2 presents SRS total score and the CCC-2 GCC according to the NVP variable. The ANCOVA conducted on the SRS total scores yielded a significant main effect of NVP group, *F*(4,277) = 4.15, *p* .003, ${\eta}_p^2$ = .057. Figure 1a illustrates how social difficulties increase as a function of more severe levels of NVP, with the linear trend yielding *t*(277) = 3.36, *p* < .001, η_p_^2^ = .039. The ANCOVA of the CCC-2 GCC data also revealed a significant main effect of the NVP group, *F*(4,204) = 2.65, *p* < .035, ${\eta}_p^2$ = .049, with lower GCC scores (i.e., greater language difficulties) associated with more severe maternal NVP (see Fig. 1b). Most of the variance in GCC scores across levels of NVP accounted for by the linear trend, *t*(204) = 3.13, *p* = .002, η_p_^2^ = .046.

**Table 2:** Means (SD, *n*) for the SRS total sore and the CCC-2 GCC as a function of the NVP variable and child sex. Effect sizes (η_p_^2^) and *p* values are based on one-way ANOVAs with NVP group as the factor

	None	Occasional nausea but no vomiting	Daily nausea but no vomiting	Occasional vomiting with or without nausea	Daily nausea and vomiting	Effect size	*p* value
SRS total^a^	99.79 (24.85, 58)	106.17 (29.48, 70)	104.28 (31.34, 54)	105.46 (27.65, 41)	119.41 (23.76, 59)	.057	.003
CCC-2 GCC^b^	33.22 (12.68, 46)	32.52 (14.14, 52)	29.68 (12.93, 40)	28.38 (13.02, 29)	24.95 (14.82, 42)	.049	.035

^a^Higher scores indicate higher levels of impairment

^b^Lower scores indicate higher levels of impairment

## Discussion

This is the first study to report the relationship between maternal experience of NVP and ASD severity in offspring. Increasing frequency and severity of NVP was positively related to the severity of social difficulties (SRS total score) and communication impairments (CCC-2 GCC scale) in offspring. The difference in the average SRS total and CCC-2 GCC scores between the offspring of mothers who experienced no NVP and those whose mothers had daily nausea and vomiting was approximately 0.8 of a standard deviation for each measure. While no study has quantified a clinically significant difference using these measures, a difference of this magnitude is highly likely to reflect clinically relevant differences in the severity of each phenotype. The cause of NVP is multifactorial and most likely involves pregnancy-related changes in the maternal hormone milieu. While the current study did not directly measure maternal hormones during pregnancy, there is considerable evidence linking the maternal experience of NVP to dysregulation of hCG, thyroid hormones, and estrogens. Several studies have reported an increased risk for offspring ASD among women experiencing dysregulation of hCG [21] and thyroid hormones [22–24] during pregnancy. The current study is the first to identify a link between NVP and the severity of ASD phenotype and provides further evidence for the possible involvement of prenatal hormone exposure in the causal pathway(s) for the ASD behavioral phenotype [3].

It is important to highlight that non-hormonal factors have also been linked to the onset of NVP. For example, twin studies have reported high heritability for NVP both within the normal range [32] and at the clinical extreme (*hyperemesis gravidarum*) [33]. A potentially fruitful line of research would be to examine how NVP may co-occur with heritable autistic traits—the so called broader autism phenotype [34]—in mothers of children with ASD. The co-occurrence of these two phenotypes at above chance levels may afford further insights into the pattern of findings identified in the current study. Furthermore, several studies have found that between 90 and 95% of women with hyperemesis gravidarum are seropositive for *Helicobacter pylori* (*H. pylori*) compared to between 45 and 50% of women who do not experience NVP [35, 36]. While these prevalence figures have not always been replicated [37], a meta-analysis of 25 case-control studies reported a pooled odds ratio of 3.32 (95% confidence interval 2.25–4.90) for *H. pylori* infection in women with hyperemesis gravidarum [38]. Unlike hormonal theories for NVP, the biological pathways linking maternal *H. pylori* infection and disrupted fetal neurodevelopment are less clear. Recent evidence from both preclinical [39] and human studies [40] suggests that maternal infection with *H. pylori* is associated with a higher risk of fetal growth restriction, which may implicate poor placental function [41]. Furthermore, numerous epidemiological studies have found that maternal *H. pylori* infection increases risk for pre-eclampsia [42, 43], potentially through negative effects on early placental development and function [44]. However, it is important to emphasize that *H. pylori* was not measured in this study and the rate of gestational hypertension and preeclampsia in the study sample was low, and thus, it was difficult to ascertain its relationship with maternal NVP. An alternative potential mechanism is the possible effect that a maternal inflammatory reaction (in response to *H. pylori* infection) may have on the developing fetus [45]. There is a strong literature describing the effects of maternal immune activation during pregnancy on fetal neurodevelopment [46], with a particular focus on the increased risk of offspring ASD [47–49]. However, no study has examined maternal *H. pylori* infection in relation to offspring ASD, and it remains unclear how bacteria that infects the stomach of half of the human population [50] may cause an inflammatory response that disrupts neurodevelopment in only a relatively small proportion of children.

Another area of growing research interest is how the sex of the fetus may influence vulnerability to differences in the prenatal environment. While the majority of research in this area has highlighted the vulnerability of male fetuses to prenatal perturbations, the effects of NVP and associated hormones on fetal outcomes has predominantly identified effects for female offspring. Numerous epidemiological studies have found that NVP is more common when gestating a female fetus [51–53], and this finding was replicated in the current study. Several studies have also reported that maternal experience of NVP [53] and increased hCG concentrations during pregnancy [54] influence growth rates in female but not male fetuses. The mechanisms by which biological sex may impart vulnerability or protection on the fetal nervous system remain largely unknown [55]. Fetal growth rate is reflective of intrauterine conditions, and fetal growth restriction is known to be associated with poor placental function [56]. There is increasing evidence that the placenta functions in a sex-specific manner and has different interactions with steroid pathways, growth factor pathways, and the fetal-placental immune system depending on the sex of the fetus [57]. While the current study did not have adequate statistical power to conduct analyses separately for male and female offspring (see Additional file 1), this background evidence suggests that an investigation of whether offspring sex moderates the relationship between NVP severity and ASD symptomatology is an important priority for future research.

A strength of the study design was the availability of standardized and widely used assessments of symptomatology on a large cohort of children with ASD that were assessed in a single geographic location (Perth, Western Australia), thus limiting bias in data collection methods. However, we note two important limitations of the study design. First, the scale used to measure NVP was developed for the purpose of this study and has not undergone broader validation. The most widely used prospective scale—the Pregnancy-Unique Quantification of Emesis and nausea (PUQE) [58]—asks women about NVP symptoms in the immediately preceding 12-h period. Given the retrospective nature of the current study, the PUQE was not considered a reliable measure of NVP, and the scale used in the current study incorporated broader NVP categories asking about daily NVP experiences (compared to hourly experiences in the PUQE). However, a major limitation of this scale is the lack of information on the timing and duration of NVP experienced by the mothers. While it is estimated that 91% of women experience NVP prior to 20 weeks’ gestation [59], there is considerable variability in the onset (and therefore duration) of symptoms. This information was not recorded in the current study, which limits the conclusions that can be drawn from the dataset. Furthermore, while the current study identified significant linear trends across NVP categories of increasing frequency and different symptomatology (nausea vs vomiting), it remains unclear whether these represent a true gradient of NVP severity. However, we note that treatment studies for NVP often differentiate between nausea and vomiting in defining NVP symptoms of increasing severity [60], and epidemiological studies have identified differential relationships between these categories and a number of pregnancy [61] and offspring [62, 63] outcomes.

A second limitation is that the obstetric information, including information on NVP, was collected via retrospective maternal report, which may be subject to a recall bias [64]. While the maternal experience of hyperemesis gravidarum is routinely noted in medical records, the full spectrum of NVP symptomatology is not prospectively recorded, which makes it challenging to determine the accuracy of maternal recall in this study. An average of 8 years had passed since the experience of NVP in the current maternal sample. While there is evidence that mothers have moderate levels of accuracy in recalling severe obstetric events (e.g., hyperemesis gravidarium) across this time period [65], there is less evidence of the validity of recall of the broader spectrum of NVP symptoms. Previous studies have also reported that a child’s health status can bias maternal recall [66], such that mothers of children with greater health or developmental difficulties may be less accurate in recalling obstetric events. While there is no evidence for this phenomenon in the ASD population, the possibility that mothers of children with more severe ASD symptoms inaccurately recalled more severe NVP symptoms than they actually experienced remains an important consideration in the interpretation of the current findings.

## Conclusion

The current study identified a positive association between increasing frequency and severity of maternal NVP and ASD severity in offspring. Based on current understanding of the biological mechanisms underpinning NVP, the findings from the current study provide further evidence that exposure to an atypical prenatal hormone environment may affect neurodevelopment and contribute to the ASD phenotype. Given this is the first such finding, replication of this effect in a separate cohort is a priority before any further conclusions can be made. Furthermore, the current study only examined NVP in pregnancies that produced an offspring who received an ASD diagnosis. Future studies can extend this work through a “within family” study design that compares the maternal experience of NVP between pregnancies from the same mother that resulted in a child with ASD and a sibling without ASD.

## Additional file

Additional file 1: Power analyses for a one-way ANOVA of the results. **Table S1.** Power analyses for a one-way ANOVA of the five-level NVP variable. **Table S2.** Means (SD, *n*) for the total and subscale scores of the SRS as a function of maternal nausea and vomiting during pregnancy and child sex. Higher scores equates to greater levels of impairment. **Table S3.** Means (SD, *n*) for the General Communication Composite and subscale standard scores of the CCC-2 as a function of maternal nausea and vomiting during pregnancy and child sex. Lower scores equate to greater levels of impairment. (DOCX 46 kb)

## Abbreviations

ASD: Autism spectrum disorder
CCC-2: Children’s Communication Checklist–2nd edition
GCC: Global Communication Composite
*H. pylori*: *Helicobacter pylori*
hCG: Human chorionic gonadotropin
NVP: Nausea and vomiting during pregnancy
SRS: Social Responsiveness Scale
