# Supplementary material for: Symptom severity in autism spectrum disorder is related to the frequency and severity of nausea and vomiting during pregnancy: a retrospective case-control study
Source: Mol Autism. 2018 Jun 19;9:37. doi: 10.1186/s13229-018-0223-7 (PMC6009817; doi:10.1186/s13229-018-0223-7)
Supplement: Supplementary file 1 — Power analyses for a one-way ANOVA of the results. Table S1. Power analyses for a one-way ANOVA of the five-level NVP variable. Table S2. Means (SD, n) for the total and subscale scores of the SRS as a function of maternal nausea and vomiting during pregnancy and child sex. Higher scores equates to greater levels of impairment. Table S3. Means (SD, n) for the General Communication Composite and subscale standard scores of the CCC-2 as a function of maternal nausea and vomiting during pregnancy and child sex. Lower scores equate to greater levels of impairment. (DOCX 46 kb) [file 13229_2018_223_MOESM1_ESM.docx]

Supplementary information

**Method**

*Power calculations*

Power calculations were conducted for the variable assessing nausea and vomiting during pregnancy (NVP) according to sex using G*Power (version 3.0.10), assuming alpha = .05 (two tailed). Additional file 1: Table S1 shows that one-way ANOVAs were well powered (>80%) to identify medium effect sizes (*f* = .25) for the combined participant sample (males and females) as well as for the male only sample. However, the female only participant sample was not adequately powered to identify large effects. For this reason, the analyses were conducted for the combined sample only.

*Table S1.* Power analyses for a one-way ANOVA of the 5-level NVP variable.

|  | Full Sample (n = 287) | Males Only (n = 227) | Females Only (n = 60) |
| --- | --- | --- | --- |
| Medium effect (*f* = .25, $\eta_{p}^{2}$ = .06) | .938 | .863 | .278 |
| Large effect (*f* = .40, $\eta_{p}^{2}$ = .14) | 1.000 | .999 | .653 |

**Results**

*Sex-specific for descriptive data for SRS and CCC-2 scores*

Additional file 1: Table S2 presents descriptive data (M, SD, n) for the SRS Total and subscales scores as a function of sex, and Additional file 1: Table S3 presents descriptive data (M, SD, n) for the CCC-2 General Communication Composite and subscale scores as a function of sex.

*Table S2.* Means (SD, n) for the Total and subscale scores of the SRS as a function of maternal nausea and vomiting during pregnancy and child sex. Higher scores equates to greater levels of impairment.

|  |  | **None** | **Occasional nausea but no vomiting** | **Daily nausea but no vomiting** | **Occasional vomiting with or without nausea** | **Daily nausea and vomiting** |
| --- | --- | --- | --- | --- | --- | --- |
| **Total score** | **Both sexes** | 99.79  (24.85, 58) | 106.17  (29.48, 70) | 104.28  (31.34, 54) | 105.46  (27.65, 41) | 119.41  (23.76, 59) |
|  | **Male** | 100.53  (24.91, 47) | 106.73  (30.66, 64) | 102.30  (31.06, 40) | 102.53  (26.43, 32) | 115.40  (22.37, 40) |
|  | **Female** | 96.64  (25.55, 11) | 100.17  (9.95, 6) | 109.93  (32.62, 14) | 115.89  (30.94, 9) | 127.84  (24.98, 19) |
| **Social**  **Awareness** | **Both sexes** | 13.56  (3.07,57) | 13.93  (3.67,70) | 13.57  (3.88,53) | 13.22  (3.85, 40) | 15.19  (3.15, 59) |
|  | **Male** | 13.54  (2.92, 46) | 14.06  (3.77, 64) | 13.41  (3.45, 39) | 13.29  (3.96, 31) | 14.78  (2.92, 40) |
|  | **Female** | 13.64  (3.78, 11) | 12.50  (2.07, 6) | 14.00  (5.01, 14) | 13.00  (3.67, 9) | 16.05  (3.52, 19) |
| **Social**  **Cognition** | **Both sexes** | 19.96  (5.00, 57) | 20.19  (6.31, 70) | 20.25  (6.17, 53) | 20.10  (5.57, 39) | 22.49  (4.83, 59) |
|  | **Male** | 20.11  (5.02, 46) | 20.28  (6.57, 64) | 19.85  (6.26, 39) | 19.47  (5.56, 30) | 21.68  (4.55, 40) |
|  | **Female** | 19.36  (5.16, 11) | 19.17  (2.14, 6) | 21.36  (5.98, 14) | 22.22  (5.38, 9) | 24.21  (5.05, 19) |
| **Social**  **Communication** | **Both sexes** | 34.09  (8.09, 57) | 36.17  (10.91, 70) | 34.89  (11.03, 53) | 35.60  (9.75, 40) | 39.86  (7.76, 59) |
|  | **Male** | 34.33  (8.24, 46) | 36.28  (11.30, 64) | 34.27  (10.77, 40) | 34.65  (9.30, 31) | 39.05  (7.90, 40) |
|  | **Female** | 33.09  (7.76, 11) | 35.00  (5.66, 6) | 36.77  (12.06, 13) | 38.89  (11.31, 9) | 41.58  (7.37, 19) |
| **Social**  **Motivation** | **Both sexes** | 14.41  (5.95, 48) | 16.09  (6.34, 70) | 15.5  (6.66, 54) | 15.95  (5.45, 41) | 18.78  (6.40, 59) |
|  | **Male** | 14.53  (5.85, 47) | 16.08  (6.60, 64) | 15.20  (6.79, 40) | 15.5  (5.25, 32) | 17.45  (6.15, 40) |
|  | **Female** | 13.91  (6.63, 11) | 16.17  (2.93, 6) | 16.36  (6.43, 14) | 17.56  (6.19, 9) | 21.58  (6.16, 19) |
| **Autistic**  **Mannerisms** | **Both sexes** | 18.21  (6.71, 58 | 19.80  (6.87, 70) | 20.74  (7.80, 54) | 20.90  (7.51, 41) | 23.25  (6.23, 59) |
|  | **Male** | 18.57  (6.61, 47) | 20.03  (7.00, 64) | 20.13  (7.67, 40) | 19.97  (7.07, 32) | 22.7  (6.06, 40) |
|  | **Female** | 16.64  (7.23, 11) | 17.33  (5.09, 6) | 22.50  (8.20, 14) | 24.22  (8.50, 9) | 24.42  (6.74, 19) |

*Table S3.* Means (SD, n) for the General Communication Composite and subscale standard scores of the CCC-2 as a function of maternal nausea and vomiting during pregnancy and child sex. Lower scores equate to greater levels of impairment.

|  |  | **None** | **Occasional nausea but no vomiting** | **Daily nausea but no vomiting** | **Occasional vomiting with or without nausea** | **Daily nausea and vomiting** |
| --- | --- | --- | --- | --- | --- | --- |
| **General Communication Composite** | **Both sexes** | 33.22  (12.68, 46) | 32.52  (14.14, 52) | 29.68  (12.93, 40) | 28.38  (13.02, 29) | 24.95  (14.82, 42) |
|  | **Male** | 32.43  (11.77, 37) | 32.33  (14.50, 48) | 32.66  (11.77, 32) | 31.77  (12.51, 22) | 25.04  (13.69, 27) |
|  | **Female** | 36.44  (16.30, 9) | 34.75  (9.85, 4) | 17.75  (10.70, 8) | 17.71  (8.28, 7) | 24.80  (17.18, 15) |
| **Speech** | **Both sexes** | 5.30  (3.91, 46) | 5.42  (3.47, 52) | 5.33  (4.24, 42) | 4.45  (3.55, 31) | 4.86  (4.16, 42) |
|  | **Male** | 5.11  (3.73, 37) | 5.33  (3.47, 48) | 5.97  (4.09, 34) | 5.13  (3.65, 23) | 4.26  (3.94, 27) |
|  | **Female** | 6.11  (4.73, 9) | 6.50  (3.70, 4) | 2.63  (3.80, 8) | 2.50  (2.51, 8) | 5.93  (4.46, 15) |
| **Syntax** | **Both sexes** | 4.22  (3.68, 46) | 3.58  (2.99, 52) | 4.41  (3.86, 42) | 3.00  (2.65, 30) | 3.14  (3.99, 42) |
|  | **Male** | 3.81  (3.26, 37) | 3.33  (2.84, 48) | 4.912  (3.94, 34) | 3.04  (2.60, 23) | 2.44  (3.89, 27) |
|  | **Female** | 5.89  (4.94, 9) | 6.50  (3.70, 4) | 2.25  (2.71, 8) | 2.86  (3.02, 7) | 4.40  (3.98, 15) |
| **Semantics** | **Both sexes** | 5.07  (2.30, 45) | 4.77  (2.16, 52) | 4.32  (2.61, 41) | 3.90  (2.35, 30) | 3.38  (2.60, 42) |
|  | **Male** | 5.17  (2.29, 36) | 4.79  (2.24, 48) | 4.76  (2.55, 33) | 4.52  (2.11, 23) | 3.52  (2.41, 27) |
|  | **Female** | 4.67  (2.45, 9) | 4.50  (0.58, 4) | 2.50  (2.14, 8) | 1.86  (2.04, 7) | 3.133  (3.00, 15) |
| **Coherence** | **Both sexes** | 3.52  (1.67, 46) | 3.69  (1.92, 52) | 3.20  (1.83, 40) | 3.21  (1.74, 29) | 2.48  (2.09, 42) |
|  | **Male** | 3.51  (1.66, 37) | 3.63  (1.95, 48) | 3.56  (1.76, 32) | 3.68  (1.64, 22) | 2.70  (2.00, 27) |
|  | **Female** | 3.44  (1.81, 9) | 4.50  (1.29, 4) | 1.75  (1.39, 8) | 1.71  (1.11, 7) | 2.07  (2.25, 15) |
| **Inappropriate Initiation** | **Both sexes** | 5.07  (2.02, 46) | 5.08  (2.59, 52) | 4.24  (1.94, 42) | 4.90  (2.40, 31) | 4.36  (2.5, 42) |
|  | **Male** | 4.97  (1.99, 37) | 5.17  (2.67, 48) | 4.47  (1.96, 34) | 5.35  (2.37, 23) | 4.55  (2.26, 27) |
|  | **Female** | 5.44  (2.19, 9) | 4.00  (0.82, 4) | 3.25  (1.58, 8) | 3.63  (2.13, 8) | 4.00  (2.80, 15) |
| **Stereotyped Language** | **Both sexes** | 4.61  (2.27, 46) | 4.10  (2.81, 51) | 3.63  (2.27, 41) | 3.79  (2.60, 29) | 2.88  (2.13, 42) |
|  | **Male** | 4.43  (2.21, 37) | 4.06  (2.86, 47) | 3.91  (2.33, 33) | 4.23  (2.67, 22) | 3.30  (2.22, 27) |
|  | **Female** | 5.33  (2.50, 9) | 4.50  (2.38, 4) | 2.5  (1.60, 8) | 2.43  (1.90, 7) | 2.13  (1.81, 15) |
| **Use of Context** | **Both sexes** | 2.67  (2.32, 46) | 3.08  (2.54, 52) | 2.42  (2.20, 41) | 2.67  (2.14, 30) | 2.05  (2.28, 42) |
|  | **Male** | 2.70  (2.16, 37) | 3.19  (2.59, 48) | 2.75  (2.26, 33) | 3.26  (2.05, 23) | 2.26  (2.33, 27) |
|  | **Female** | 2.56  (3.05, 9) | 1.75  (1.26, 4) | 1.00  (1.12, 8) | 0.71  (0.95, 7) | 1.67  (2.23, 15) |
| **Nonverbal Communication** | **Both sexes** | 2.57  (1.73, 46) | 2.51  (1.64, 51) | 2.46  (1.50, 41) | 2.32  (1.66, 31) | 1.81  (1.44, 42) |
|  | **Male** | 2.46  (1.61, 37) | 2.51  (1.71, 47) | 2.61  (1.39, 33) | 2.67  (1.58, 23) | 2.00  (1.36, 27) |
|  | **Female** | 3.00  (2.24, 9) | 2.50  (0.58, 4) | 1.88  (1.885, 8) | 1.25  (1.49, 8) | 1.47  (1.55, 15) |
| **Social Relations** | **Both sexes** | 2.65  (2.17, 46) | 2.35  (2.34, 52) | 1.91  (2.05, 42) | 1.68  (1.89, 31) | 1.15  (1.42, 42) |
|  | **Male** | 2.65  (2.23, 37) | 2.38  (2.41, 48) | 2.17  (2.14, 34) | 2.04  (1.92, 23) | 1.42  (1.36, 26) |
|  | **Female** | 2.67  (2.06, 9) | 2.00  (1.41, 4) | 0.75  (1.04, 8) | 0.63  (1.41, 8) | 0.67  (1.45, 15) |
| **Interests** | **Both sexes** | 4.96  (2.25, 46) | 4.84  (2.49, 50) | 4.07  (2.36, 42) | 4.03  (2.37, 31) | 3.81  (2.44, 42) |
|  | **Male** | 4.83  (2.09, 37) | 4.91  (2.57, 46) | 4.29  (2.51, 34) | 4.39  (2.33, 23) | 4.00  (2.30, 27) |
|  | **Female** | 5.44  (2.92, 9) | 4.00  (0.82, 4) | 3.13  (1.36, 8) | 3.00  (2.33, 8) | 3.47  (2.72, 15) |
